# Migrant status disparities in blood pressure: a multiple mediation analysis of modifiable factors

**DOI:** 10.1007/s44197-023-00136-x

**Published:** 2023-07-08

**Authors:** Teresa Dalla Zuanna, Erich Batzella, Francesca Russo, Gisella Pitter, Cristina Canova

**Affiliations:** 1 Unit of Biostatistics, Epidemiology and Public Health, Department of Cardio-Thoraco-Vascular Sciences and Public Health, University of Padua, Via Loredan 18, 35100 Padua, Italy; 2 Directorate of Prevention, Food Safety, and Veterinary Public Health-Veneto Region, Venice, Italy; 3 Screening and Health Impact Assessment Unit, Azienda Zero-Veneto Region, Padua, Italy

**Keywords:** Blood pressure, Hypertension, Immigrants, Mediation, Body mass index

## Abstract

**Background:**

We examined differences in blood pressure (BP) levels between first-generation immigrants and natives in adult residents in Northeast Italy, and investigated the role of lifestyle behaviors, body mass index (BMI), and education as potentially modifiable mediating factors.

**Methods:**

We included 20–69-year-old participants from the Health Surveillance Program of the Veneto Region (*n* = 37,710). Immigrants born in a high migratory pressure country (HMPC) were further grouped into geographical macro-areas. The outcomes were systolic BP (SBP) and hypertension. Multiple mediation analyses were performed to determine the contribution of each mediator of the SBP/migrant status association.

**Results:**

Of the 37,380 subjects included, 8.7% were born in an HMPC. BMI, education, alcohol, sweets and meat consumption were included as potential mediators. A small advantage in SBP was seen for immigrants compared to natives (β =  – 0.71,95%CI  – 1.30;  – 0.10). The direct effect (net of the covariates) of immigrant status on SBP was a reduction of 1.62 mmHg (95%CI  – 2.25;  – 0.98). BMI played the highest suppressive role (β = 1.14,95%CI 0.99; 1.35), followed by education. Alcohol consumption amplified the health advantage of immigrants. The suppressing effect of BMI was particularly evident among women and North Africans compared to natives. Similar results were seen for hypertension rates.

**Conclusions:**

Although causation cannot be proven given the cross-sectional design, our findings identify BMI as the most effective target to preserve the health advantage of immigrants with respect to BP levels.

**Supplementary Information:**

The online version contains supplementary material available at 10.1007/s44197-023-00136-x.

## Introduction

Elevated blood pressure (BP) is the leading global risk factor for cardiovascular diseases [1]. There is a continuous linear relationship between BP levels and the risk of stroke or myocardial infarction, and treatments to lower BP provide significant protection against CV events [2]. Over the past four decades, the rates of increased systolic BP (SBP ≥ 140 mm Hg) have increased substantially, particularly in low- and middle-income countries [3].

Disparities in BP values and in hypertension-related diseases persist in most countries among different ethnic groups or migrant groups compared to natives [4–6]. A large meta-analysis on differences in BP in Europe showed higher levels in immigrants from Sub-Saharan Africa and lower levels in the Muslim population compared to the native population [7]. Our recent study conducted in northeastern Italy found no differences in BP levels between Italians and immigrants; these effects were robust when examining the overall group of immigrants and when stratified by macro-area of origin [8]. When adjusting the results for a set of potential covariates (lifestyle factors and social determinants), an advantage was observed for the immigrants compared to Italians. This result suggests that some covariates may act as potential mediators in the relationship between migrant status and BP level, with an effect similar in magnitude but opposite sign, thus acting as a suppressor of this association [9].

Different factors have been studied as possible mediators of the disparities in BP levels [5, 10]. Racial disparities and disparities by migrant status have been found to be modified by educational level [11, 12] and by behavioral [13] and clinical factors, such as body mass index (BMI) level [13–15]. It is essential to identify which (and in what amounts) of these modifiable mediators intervene in the association of migrant status and BP, to improve risk stratification and guide the development of effective interventions. Furthermore, since clinical alterations such as raised BP are detectable before the onset of the disease itself, the analysis of its mechanisms of emergence could help detect health differences earlier than the analyses on the confirmed hypertensive diseases or hypertension-mediated complications. Therefore, the aim of this study is to examine differences in BP levels between first-generation immigrants and native-born Italians in a large population of adult residents in Northeast Italy and to explore the multiple factors that can explain the disparity by migrant status in BP.

## Methods

### Participants and study design

Data were retrieved from a publicly funded health surveillance program implemented by the Veneto Region in 30 municipalities located in Northeast Italy [16]. This program is a population-based screening started in 2017 with the aim of prevention, early diagnosis, and treatment of chronic disorders possibly associated with the high perfluoroalkyl substance exposure discovered in this area in 2013. A detailed description of the program can be found elsewhere [16]. Surveillance involved the active calling of the eligible population and the free offer of health examinations, including (i) a questionnaire on sociodemographic characteristics, health history, diet and lifestyle characteristics, self-reported height and weight; (ii) measurement of BP; and (iii) blood and urine samples.

All subjects aged 20–69 years recruited until May 2021 were included in this cross-sectional analysis (*n* = 38,292, participation rate 61%). Pregnant women and participants with missing data on relevant variables were excluded, leaving a total of 37,710 subjects in the analysis (Figure S1).

### Exposure: country of birth

Immigrant status was defined by the self-reported country of birth. Italian-born residents were compared to immigrants born in high migratory pressure countries (HMPC), further grouped into 5 geographical macro-areas: Central-Eastern (CE) Europe, Central and Southern (CS) America, North Africa, Sub-Saharan (SS) Africa and Asia (except for Israel and Japan)[17]. Immigrants born in low migratory pressure countries were a very small proportion of the study population (*n* = 330, 0.87%) and were excluded from the analyses, leaving 37,380 subjects (Figure S1).

### Outcomes: SBP and hypertension

BP was measured by trained health nurses with participants first sitting at rest for at least 5 min, according to the European Society of Hypertension recommendations [18]. A validated semiautomatic sphygmomanometer with an appropriate cuff size for the arm circumference was used.

The main analysis investigated the SBP values as a continuous variable in 32,521 participants, excluding self-reported diagnosis of hypertension or under treatment with anti-hypertensive drugs (*n* = 4,859) (Figure S1). We chose the SBP level as the only continuous outcome, because it is the major risk factor for cardiovascular disease [19].

As a secondary outcome, we focused on hypertension prevalence, which is defined as SBP ≥ 140 mmHg or diastolic BP ≥ 90 mmHg, self-reported diagnosis of hypertension, or current use of antihypertensive medications (*n* = 9,353).

### Statistical analysis

**Fig. 1:**
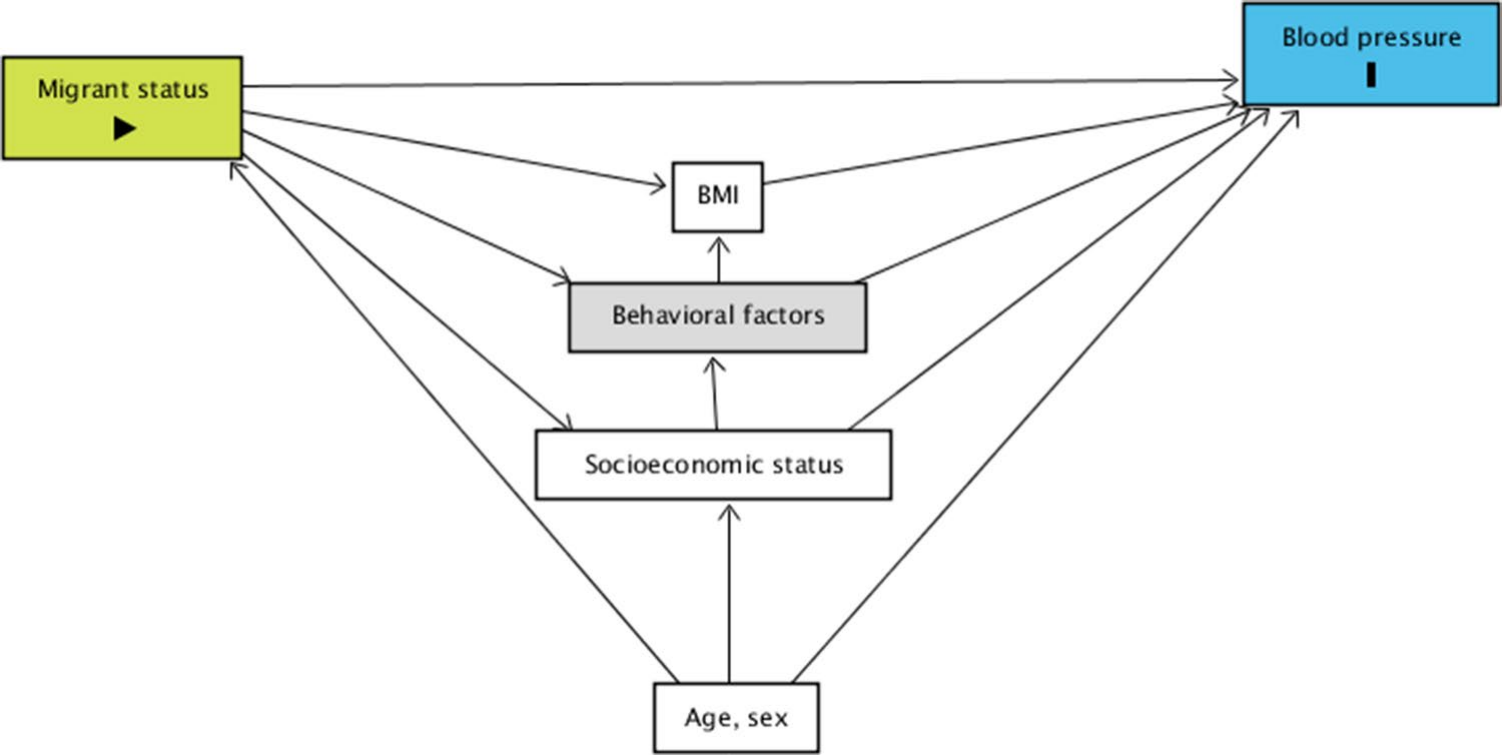
Path diagram of the association of migrant status and blood pressure

Covariates to be included as potential mediators or confounders of the SBP/migrant status association were selected from the available variables based on related literature, and a path diagram of mediation/confounding effects was developed. This conceptual framework (Fig. 1) illustrates the associations of migrant status with SBP level and hypertension, the possible mediating roles of educational level, lifestyle factors and BMI, and the potential confounding effects of age and sex. Educational level was divided into primary/middle school, high school, university or higher degree. Lifestyle factors considered were alcohol consumption (0, 1–2, 3 + alcohol units per week) and smoking status (current smokers, previous smokers/nonsmokers), the diet components known to be associated with raised BP (salt consumption divided into low, medium and high consumption, sweets and meat consumption in tertiles of consumption[20]). BMI (kg/m^2^) was determined based on self-reported height and weight.

The selected covariates illustrated in the diagram (Fig. 1) are potential mediators of the relationship between migrant status and the selected outcomes. A mediator is defined as a variable that is on the causal pathway between the predictor and the outcome of interest. Therefore, a variable is considered a potential mediator if: (1) the potential mediating factor is distributed differently among the population groups; and (2) the potential mediating factor is significantly associated with the outcomes. We used t tests and chi-square tests to assess the association between migrant status and each covariate (Condition 1). We assessed the association of each covariate and the two outcomes (Condition 2) using generalized linear models adjusting for age and sex. A likelihood ratio test (LRT) was used to test the significance of each possible mediator.

We then performed multiple mediation analysis using the method based on the counterfactual framework proposed by Yu and Li [21], which allowed us to include factors measured in different ways (continuous, binary or categorical) and to account for the correlations among factors. The method was implemented using the mma package in the statistical software R and was explained in detail elsewhere [22]. We used a multiple generalized linear model (logistic regression for hypertension) to calculate the total direct effect and the total indirect effect. The total effect represents the overall effect of being an immigrant compared to natives on SBP levels. The direct effect indicates the size of the effect not explained by any of the mediating factors. The indirect effect indicates the size of the effect explained by the mediating factors. In addition, the indirect effect of each individual mediator in the relationship of migrant status and SBP levels/hypertension prevalence was determined.

Confidence intervals were computed based on the estimated mediation effects from bootstrap samples. We decided not to compute the relative effects, defined as the ratio of the corresponding (in)direct effect over the total effect, because they are hardly reasonable and interpretable when the direct and indirect effects have opposite signs [23].

All analyses were stratified by sex and by the three macro-areas with sufficient sample sizes (CE Europe, North Africa, Asia). We also decided to perform a sensitivity analysis including all available mediators in the model, even those not satisfying the preliminary conditions.

### Ethics approval

The study was approved by the Regional (Veneto Region) Ethics Committee (May, 24 2017; prot. n. 203,638). All participants provided written informed consent.

## Results

### Baseline characteristics and association of the exposure with the potential mediators

Overall, 37,380 subjects were included in the analysis, 3249 (8.7%) of whom were born in an HMPC (Figure S1). Immigrant males were older, while females were younger than natives. Immigrants had higher BMI values (especially females), lower rates of smoking, higher salt consumption but lower rates of meat, sweets and alcohol consumption. In addition, immigrants had a lower educational level than their Italian counterparts. SBP levels were higher among Italians than immigrants (mean (SD) = 121.8 (14.7) mmHg vs 120.5 (14.6) mmHg), as was the prevalence of hypertension (25.3% vs 21.9%) (Table S1). All tests performed to compare covariate distributions between immigrants and natives showed *p* values below 0.05, except for meat consumption among females (*p* value = 0.08). Baseline characteristics of the study population by the three most representative macro-areas of origin are presented in Table S2. The hypertension prevalence varied greatly among immigrants in relation to the macro-area of origin: immigrants from CE Europe had the highest prevalence (23.3%), followed by those from Asia (22.0%), and those from North Africa (16.6%) (Table S2).

### Associations with the outcome

The analysis of the effects of the potential mediators on the outcomes revealed important associations, as shown in Table 1. In the models adjusted for age and sex, SBP increased by 0.87 mmHg per increase in BMI (95% CI 0.83;0.90, LRT *p* value < 0.001). In addition, the SBP levels were increased by the assumption of at least one alcoholic unit per week (LRT *p* value < 0.001), the increase in sweets (LRT *p* value < 0.001) and meat consumption (LRT *p* value = 0.002). Finally, the less educated groups had higher SBP levels than the graduated group (high school vs university: β = 1.38, 95% CI 1.27;1.49. Elementary/middle school vs university: β = 1.52, 95% CI 1.40;1.65) (Table 1). There were no significant associations of smoking habits and salt consumption with SBP. There was no association between migrant status and SBP in the partially adjusted model (β =  – 0.46, 95% CI  – 0.99;0.07), and when the model was adjusted for all covariates, immigrants had an SBP level that was 1.55 mmHg lower than that of natives (95% CI  – 2.07;  – 1.02).

**Table 1:** Analyses of factors associated with the outcome (a significant increase in SBP, and prevalence of hypertension), adjusted for age and sex

Characteristics		Systolic BP	*p*-value likelihood ratio test	Hypertension	*p*-value likelihood ratio test
		β (95% CI)		OR (95% CI)	
Country of birth	*HMPC vs Italian*	– 0.46 ( – 0.99; 0.07)	0,09	0.96 (0.88; 1.06)	0,434
BMI (continuous)*	0.87 (0.83; 0.90)	0,000	1.16 (1.15; 1.17)	0,000	
BMI categorical	*Overweight vs normal weight*	4.50 (4.16; 4.84)	0,000	2.21 (2.08; 2.35)	0,000
	*Obese vs normal weight*	10.12 (9.63; 10.61)		5.68 (5.29; 6.11)	
Smoking Habit	*Current smoker vs Non-smoker /previous smokers*	0.04 ( – 0.32; 0.41)	0,815	1.06 (0.99; 1.13)	0,076
Alcohol intake*	*1–2 vs None*	0.75 (0.36; 1.13)	0,000	0.99 (0.93; 1.06)	0,070
	*3* + *vs None*	0.75 (0.35; 1.15)		1.06 (0.99; 1.13)	
Education*	*Highschool vs University*	0.94 (0.54; 1.35)	0,000	1.38 (1.27; 1.49)	0,000
	*Elementary/Middle School vs University*	1.74 (1.28; 2.20)		1.52 (1.40; 1.65)	
Salt	*Medium vs Low*	0.22 ( – 0.09; 0.53)	0,366	1.01 (0.95; 1.06)	0,074
	*High vs Low*	– 0.01 ( – 0.68; 0.66)		1.13 (1.02; 1.26)	
Sweets*	*2 vs 1*	0.42 (0.04; 0.80)	0,000	0.89 (0.83; 0.94)	0,001
	*3 vs 1*	0.93 (0.57; 1.29)		0.98 (0.92; 1.03)	
Meat*	*2 vs 1*	1.33 (0.91; 1.75)	0,002	1.11 (1.04; 1.20)	0,000
	*3 vs 1*	1.78 (1.44; 2.12)		1.25 (1.18; 1.32)	

*variable selected as potential mediator (*p* < 0.05); alcohol was forced in as potential mediators in the analysis with hypertension as outcome

Similar associations were seen with hypertension prevalence for BMI, education, sweet and meat consumption. No association was observed between smoking habits, alcohol consumption, salt consumption and the prevalence of hypertension. The partially adjusted model did not show a significant association between migrant status and hypertension (OR = 0.96, 95% CI 0.88;1.06), while in the fully adjusted model, immigrants had a 22% lower risk of hypertension than natives (OR = 0.78, 95% CI 0.71;0.86).

### Mediation analysis

All potential mediating factors were associated with exposure (Condition 1). However, smoking habits and salt consumption did not satisfy the second condition and were excluded from the main analysis. Although alcohol consumption was not significantly associated with hypertension (*p* = 0.07), it was forced in the analysis as a potential mediator, because it is known to be associated with SBP levels.

**Fig. 2:**
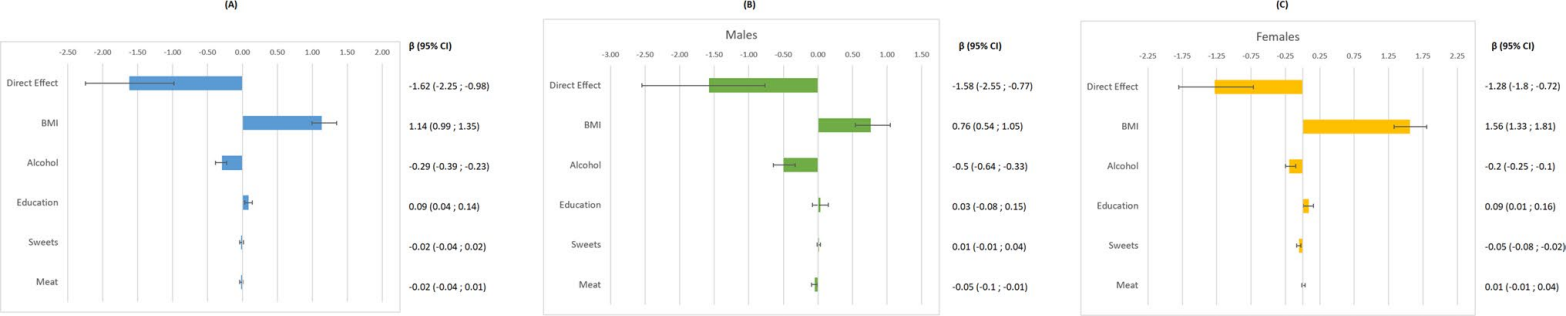
Multiple mediation analysis for HMPC vs Italy, stratified by gender. βs and corresponding 95% Confidence Intervals in all subjects (**A**), Males (**B**), and Females (**C**)

The estimates of the mediation analysis, overall and stratified by sex, are presented in Table 2 and Fig. 2. The total effect of migrant status on SBP was a reduction of 0.71 mmHg (95% CI  – 1.30;  – 0.10), that is, a rather small advantage of BP levels among immigrants compared to natives. The direct effect, obtained by removing the effect of the potential mediators, was  – 1.62 mmHg (95% CI  – 2.25;  – 0.98), showing an enhanced advantage among immigrants. Such an increase reveals that the mediators act in the opposite direction compared to the direct effect of the exposure, reducing the advantage in SBP levels that immigrants would have compared to Italians if the mediators were equally distributed across the population groups. Indeed, the total indirect effect was 0.91 mmHg (95% CI 0.72; 1.13), thus resulting in a partial suppression of the effect of migrant status on SBP levels. When the results were stratified by sex, immigrant males had an average SBP level 1.27 mmHg lower than their Italian counterpart (β =  – 1.27, 95% CI  – 2.25;  – 0.40), while females had no statistically significant disparities in SBP levels by immigrant group (β = 0.10, 95% CI  – 0.46; 0.82) (Table 2).

**Table 2:** Multiple mediation analysis for HMPC vs Italy, stratified by gender

	Systolic BP			Hypertension		
	Total	Males	Females	Total	Males	Females
	β (95% CI)	β (95% CI)	β (95% CI)	OR (95% CI)	OR (95% CI)	OR (95% CI)
Total effect	– 0.71 ( – 1.30; – 0.10)	– 1.27 ( – 2.25; – 0.40)	0.10 ( – 0.46; 0.82)	0.92 (0.83; 1.02)	0.79 (0.68; 0.94)	1.15 (1.01; 1.27)
Total direct effect	– 1.62 ( – 2.25; -0.98)	– 1.58 ( – 2.55; – 0.77)	– 1.28 ( – 1.80; -0.72)	0.78 (0.71; 0.86)	0.75 (0.64; 0.90)	0.87 (0.76; 0.97)
Total indirect effect	0.91 (0.72; 1.13)	0.31 (0.02; 0.56)	1.38 (1.20; 1.67)	1.19 (1.14; 1.22)	1.05 (0.99; 1.11)	1.32 (1.26; 1.38)

Of the potential mediators, only BMI, alcohol intake and education reached a statistically significant indirect effect. In particular, the indirect effect of BMI is greater than the total indirect effect itself, meaning that if there was no direct effect and no other mediators, immigrants would have an average higher SBP of 1.14 mmHg compared to natives (95% CI 0.99;1.35) due to the differences in the BMI distribution. The educational level also played a similar role, but much smaller in magnitude (β = 0.09, 95% CI 0.04; 0.14), while the lower alcohol intake of the immigrant group was a protective factor for the immigrant group, having a negative indirect effect (β =  – 0.29 95% CI  – 0.39;  – 0.23) (Fig. 2a). In the analyses stratified by sex, the BMI increased SBP levels by an average of 1.56 mmHg for immigrant females compared to natives (95% CI 1.33; 1.81) (Fig. 2b), while among males, BMI increased BP by only 0.76 mmHg (95% CI 0.54; 1.05). In addition, the negative indirect effect of alcohol consumption is stronger among males, explaining an average reduction of 0.50 mmHg (95% CI  – 0.64;  – 0.33) compared to their Italian counterparts (Fig. 2c).

### Mediation analysis stratified by macro-area of origin

The results stratified by the macro-areas of origin (presented in Table S3) showed a similar pattern compared to the overall results: a null total effect, explained by a negative direct effect and a positive indirect effect of the immigrant population compared to natives. Differences emerged when analyzing the individual indirect effects. The BMI positive indirect effect doubled for the group from North Africa compared to the other macro-areas (β = 2.11, 95% CI 1.8; 2.59). Education level had a positive indirect effect for immigrants from Asia, CE Europe and, to a lesser extent, North Africa compared to natives. The different alcohol consumption led to a reduction in the SBP levels of immigrants from Asia and North Africa compared to natives, while it had a null effect when considering immigrants from CE Europe. Finally, the indirect effects of sweets and meat consumption reached a significant level for the Asian population.

### Hypertension

We found no differences in the odds of having hypertension among immigrants and natives (OR = 0.92, 95% CI 0.83; 1.02). When excluding the effect of potential mediators, immigrants had a 22% lower risk of being diagnosed with hypertension than natives (OR = 0.78, 95% IC: 0.71; 0.86). The total indirect effect was in the opposite direction (OR = 1.19, 95% CI 1.14; 1.22), neutralizing the otherwise health advantage of immigrants compared to natives (Table 2). The significant mediators for this outcome were the same for SBP levels, and the order of mediating effects was roughly the same (Figure S2). The results stratified by macro area of origin for the hypertension outcome are shown in Table S3.

### Sensitivity analysis

The results of the sensitivity analysis, which included all the covariates as potential mediators, are shown in Tables S4 and S5. The indirect effects of salt consumption and smoking habits, which had been added as additional potential mediators, did not reach statistical significance or generated a deviation of less than 0.05 mmHg in SBP levels.

## Discussion

Overall, we found that the association of migrant status with SBP levels and hypertension rates in our population can be unraveled in two effects, similar in magnitude, but with opposite signs: a negative direct effect (net of the selected mediators), which would result in a health advantage for immigrants, and a positive indirect effect, explained by the potential mediators, which played an unfavorable role for the immigrant group compared to natives.

The primary objective of a mediation analysis is to identify variables that intervene in the observed relationship between an exposure and a response variable or to measure the effect of an intervening variable on the relationship between an exposure and a response variable (as shown in Fig. 1). According to the counterfactual framework proposed by Yu and Li [21], only variables that exhibit significant associations with both the predictor and the outcome are considered as potential mediators, measuring their impact on the observed relationship between migrant status and SBP levels and hypertension rates. Consistent with our hypotheses, all variables included in the theoretical model, based on related literature and available information, demonstrated associations with migrant status (fulfilling the first condition). Furthermore, the majority of these variables were significantly related to the outcome (fulfilling the second condition), except for smoking habits and salt consumption.

By analyzing the effects of each mediator, we can assume that the suppressive role towards the association between SBP and migrant status was almost completely played by BMI. In other words, the BMI distribution was so unfavorable for the immigrant population that it suppressed the health advantage that immigrants would otherwise have had regarding BP levels. The role of BMI was more evident among women and immigrants from North Africa.

To our knowledge, no studies analyzed the mediators of disparities in BP levels by immigrant status, and only one study from the US has analyzed the potential mediators (1 at a time) of the racial difference in hypertension incidence [24]. In this study, the dietary pattern was the largest mediating factor for differences in the incidence of hypertension between black and white individuals. Because the mean BMI was similar among black and white men, it was not a mediating factor for the excess risk of hypertension among black men. Among black women, instead, BMI was the second largest mediator for hypertension [24]. Other studies have identified BMI as a determinant of the effects of ethnic differences or immigrant status differences on BP levels, although they considered it an adjustment variable rather than a mediator and concluded that BMI could only partially explain the observed ethnic differences in hypertension prevalence [5, 10].

Elevated BMI is associated with an increased risk of hypertension due to the extra load placed on the myocardium because of increased cardiac contractibility, which leads to increased heart rate and stroke volume. Therefore, by increasing cardiac output, BP is subject to elevation [5]. In addition, two studies conducted in the US, found that people with Asian ethnicity had higher hypertension rates than other racial/ethnic groups at the same BMI levels, suggesting that the relation between BMI and BP level could be influenced by a different genetic background and that increases in weight are more detrimental than in other ethnic groups [25, 26].

A smaller effect in mediating this association has been found for educational level and alcohol intake. A lower mean educational level among immigrants resulted in a suppression of the health advantage of immigrants. It is known that lower education is associated with higher levels of SBP and a higher hypertension prevalence [27], and in our study, this effect has been accounted for by other different risky behaviors or the BMI distribution, also known to be associated with lower education [28]. An explanation could be that the lower educational level could bring to lower access to health services. Among immigrants, lower education could be related to lower language skills, lower adaptability to a different culture, and consequently higher barriers to accessing health services. A low educational level could also be related to worse working conditions. Psychosocial factors related to physical working conditions, job strain and job control have been demonstrated to explain socioeconomic inequalities in health [29] and, more specifically, in cardiovascular outcomes [30].

Regarding alcohol intake, the lower average alcohol consumption of immigrants contributed to reducing SBP levels among immigrants compared to natives. This was particularly evident among males (except those born in CE Europe), due to the higher rates of Italian-born males drinking alcohol. Virtuous behavior still present in immigrant population should be encouraged and maintained to prevent the effect of acculturation, which can lead to an increase in alcohol consumption [31].

Other potential mediators, such as salt consumption and diet components, had no effect on the relationship between immigrant status and BP. It should be noted that these variables are self-reported in our study, and we could not rely on more objective data, such as 24-h urinary sodium excretion.

### Results by macro-area of origin

The effect of BMI previously described was higher among immigrants from North Africa, who in our study are mostly represented by subjects born in Morocco (96%, see Table S6). Moroccans have been found to have a lower prevalence of hypertension in Europe [4, 10], suggestive of a lower susceptibility [10] and an effect of behavioral habits related to the Muslim religion, highly prevalent in Moroccans, that are protective against the development of hypertension [7]. The high negative indirect effect of alcohol consumption in this population can be similarly explained. Nevertheless, this health advantage seems to be changing unfavorably through the acculturation process [4], and the indirect effect of high BMI found in our study could depend on the adoption of a westernized diet and a sedentary lifestyle.

The indirect effects of individual mediators for immigrants from CE Europe compared to Italians are small in magnitude, with the exception of the effect of BMI, which is smaller than that of Asians and North Africans. This may reflect the higher proximity of these immigrants—mainly Romanians Albanians, Serbs and Moldavians (Table S6)—to Italian habits and culture, both in a positive way (the lower effect of the differential educational level) and in a negative way (the absence of a protective effect of the alcohol consumption).

In our study, 85% of the Asian population was born in India and Bangladesh (Table S6). Immigrants from these countries to the UK have been found to have lower levels of BP among adults than their English counterpart, while higher levels of BP were registered among children (namely, the second generation immigrants) [32]. This intergenerational change in BP difference mirrored the change in BMI difference, thus confirming once again the risks of the acculturation process in the adoption of unhealthy lifestyles.

### The direct effect of migrant status on BP

In light of the findings described thus far, it must be noted that the purpose of this approach is not to exhaustively define all factors that underlie BP levels but rather to explore the existence of modifiable factors that mediate known disparities between migrants and natives. The total effect between “migrant status” and BP captures the joint effect of all factors related to the immigrant background, regardless of their modifiability [33], and the indirect effect represents the attempt to identify the specific components intrinsic to the “immigrant status” for which interventions are more conceivable than for the immigrant status itself. Otherwise, the “direct effect” of our study must be interpreted as all the other residual factors included in the fact of being an immigrant, namely, the genetic differences (that may also explain their increased BMI), the influence of the life in the country of origin, the immigration history, and related psychosocial stress. When considering this component, a health advantage appears. This result probably mainly reflects the healthy migrant effect [34], still present in this population of first-generation immigrants.

### Health interventions

This paper reflects the importance of acting on modifiable factors to gain a health advantage in BP levels among immigrants. This is particularly important given that the acculturation process, the ageing of the migrant population, and the risk of experiencing racism could lead to an increase in SBP levels and hypertension prevalence [4, 7, 35]. Finally, efforts should be made to reduce the prevalence of hypertension, because levels of hypertension awareness, treatment and control differ between ethnic minorities and Europeans [6], with the risk of higher hypertension-mediated complications among them.

Many health interventions, mainly in primary care settings, have been demonstrated to be effective for the early prevention of cardiovascular diseases. In particular, the higher BMI of the immigrant population could be reduced with interventions on nutritional habits and physical activity. In addition, it must be noted that the heterogeneous results among sexes and macro-areas indicate the need for targeted intervention towards immigrant groups with higher effects for each mediating factor.

### Strengths and limitations

The globally observed disparities by migrant status in hypertension prevalence is subject of ongoing debate, and little is known about the contribution of lifestyle, psychosocial and socio-economic determinants to the observed differences in BP levels and hypertension prevalence [10]. To our knowledge, this is the first study analyzing the potential multiple mediators of the effects of migrant status on BP. It was conducted on a large number of individuals and accounted for a wide number of potential mediators. Furthermore, this work attempts to disentangle the effect of migrant status on BP by identifying modifiable mediators that can result in feasible opportunities to implement policies to overcome disparities.

The main limitation is its cross-sectional design, and as such, we were unable to determine the temporality between the occurrence of our exposure and the mediating effect of covariates and outcomes. Anyway, examining mediation hypotheses with cross-sectional data may be reasonable if timing is implied by the nature of their construction. In our case, it is unlikely that the migration status has been influenced by lifestyle habits, especially those reported in the host country. In addition, while it is possible that individuals with hypertension may modify their lifestyle in accordance with health advice, leading to subsequent reductions in their BMI, it is important to note that continuous blood pressure values have limited influence on lifestyle choices or BMI.The purpose of this study is not to identify a causal relationship but to explore and unravel the association of BP levels and migrant status and to identify variables that can explain the disparity. However, the inferences for the two concepts are statistically identical [9]. Second, it is important to consider the potential influence of unmeasured mediators, which refer to modifiable habits within the causal pathway under investigation. In the Health Surveillance Plan, the information on occupation was often not reported or hardly categorizable, leading us to exclude it from the analysis. Similarly, we did not consider physical activity due to challenges in harmonizing this variable, which was collected differently in two consecutive versions of the interview. Previous research has indicated that individuals from ethnic minorities in Europe tend to be less physically active and achieve lower levels of physical activities [10]. Consequently, including this variable in our model might have had a similar suppression effect of the BMI, increasing the absolute value of the direct effect. However, the magnitude of this effect remains unknown. Notably, a study conducted in the US examining the impact of individual mediators on racial disparities in BP levels, found no statistically significant role played by the physical activity [24]. In addition, we could not take into account material and psychosocial factors, such as negative life events, family conflicts, or financial difficulties, which are possible mediators of the association between migrant status and occurrence of cardiovascular diseases. Third, the questionnaire was in Italian, so there might be some measurement errors of some lifestyle variables that might have been misunderstood or wrongly interpreted by migrants. The questionnaire was administered by trained nurses, so the nurses could have mediated some language barriers by explaining the questions. Furthermore, when communication was hindered by language problems, the interviewed subject was invited to return a second time with an interpreter. Finally, as our study specifically focused on comparing HMPC and Italian subjects, it is important to acknowledge that the generalizability of our findings to other populations may be limited.

## Conclusions

This paper provides an overview of modifiable factors associated with disparities by migrant status in SBP levels. Although not generalizable, our results suggest that BMI and, to a lesser extent, education and alcohol consumption are independent mediators in the relationship between migrant status and BP level. Public health measures aimed at preserving the health advantage of immigrants by targeting these factors, taking into account the specificities of each migrant group, are strongly encouraged.

## Supplementary Information

Below is the link to the electronic supplementary material.

Supplementary file1 (DOCX 127 KB)

## Data Availability

The data are not publicly available. The data presented in this study are available from the corresponding author on reasonable request.

## Abbreviations

BP: Blood pressure
BMI: Body mass index
CE: Central-Eastern
CS: Central-Southern
HMPC: High migratory pressure country
LRT: Likelihood ratio test
SBP: Systolic blood pressure
SS: Sub-Saharan
