# Supplementary material for: Migrant status disparities in blood pressure: a multiple mediation analysis of modifiable factors
Source: J Epidemiol Glob Health. 2023 Jul 8;13(3):547–56. doi: 10.1007/s44197-023-00136-x (PMC10468480; doi:10.1007/s44197-023-00136-x)
Supplement: Supplementary file 1 — Supplementary file1 (DOCX 127 KB) [file 44197_2023_136_MOESM1_ESM.docx]

Supplementary Figure 1. Flowchart of the study population.


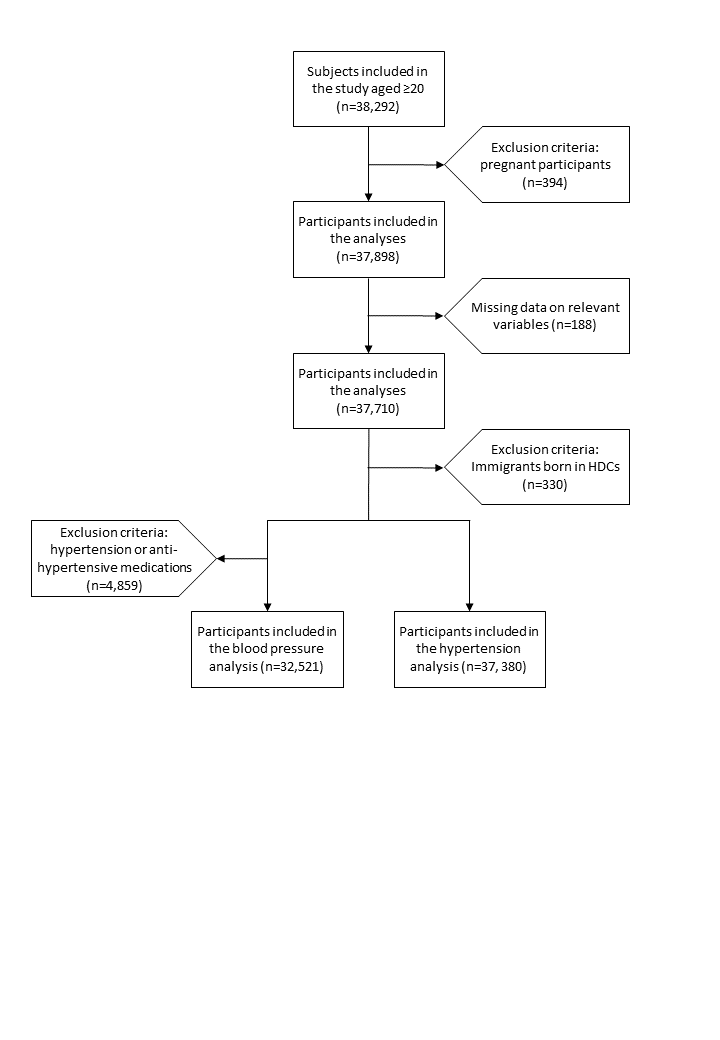


Supplementary Figure 2 Multiple mediation analysis for hypertension, HMPC vs Italy, stratified by gender. ORs and corresponding 95% Confidence Intervals in all subjects (A), Males (B), and Females (C).


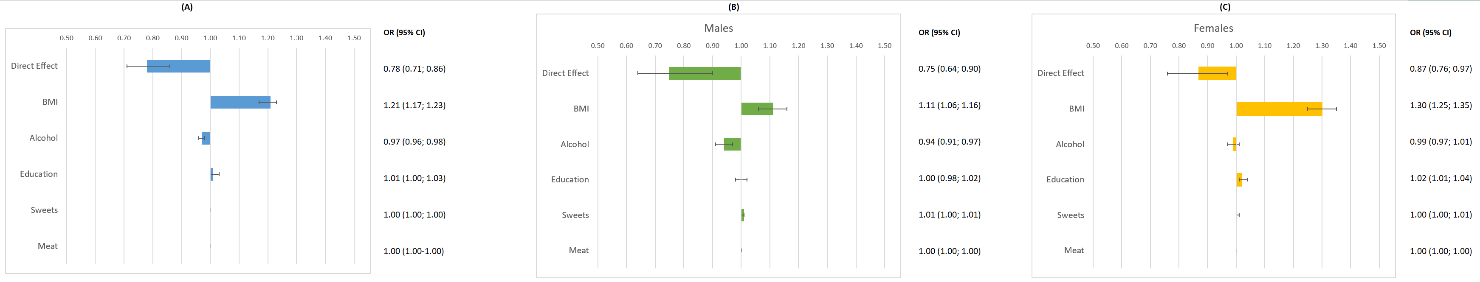


Table S1. Characteristics of the included population, stratified by migrant status (Italian vs HMPC) and by gender

| **Characteristics (n=32,521)** | | **Total** | | | | | | **Males** | | | | | **Females** | | | | |
| --- | --- | --- | --- | --- | --- | --- | --- | --- | --- | --- | --- | --- | --- | --- | --- | --- | --- |
|  |  | **ITALY (n=29,644)** | | | **HMPC (n=2,877)** | | | **ITALY (n=13,954)** | | | **HMPC (n=1,138)** | | **ITALY (n=15,690)** | | | **HMPC (n=1,739)** | |
| **continuous variables** | | *Mean (SD)* | *Min-Max* | *Mean (SD)* | | *Min-Max* | *Mean (SD)* | | *Min-Max* | *Mean (SD)* | | *Min-Max* | | *Mean (SD)* | *Min-Max* | *Mean (SD)* | *Min-Max* |
| Age (years) | | 39.8 (11.5) | 20; 67 | 39.2 (9.6) | | 20; 66 | 39.2 (11.5) | | 20; 67 | 40.0 (10.0) | | 20; 65 | | 40.3 (11.5) | 20; 67 | 38.7 (9.2) | 20; 66 |
| BMI (kg/m2) | | 24.4 (4.3) | 13.4; 54.3 | 25.75 (4.61) | | 15.6; 52.6 | 25.4 (3.8) | | 13.4; 54.3 | 26.2 (4.0) | | 16.2; 45.7 | | 23.6 (4.6) | 14. ; 54.1 | 25.5 (5.0) | 15.6; 52.6 |
| **categorical variables** | | *N* | *%* | *N* | | *%* | *N* | | *%* | *N* | | *%* | | *N* | *%* | *N* | *%* |
| BMI | *Normal weight* | 18350 | 61,9% | 1386 | | 48,2% | 7217 | | 51,7% | 478 | | 42,0% | | 11133 | 71,0% | 908 | 52,2% |
|  | *Overweight* | 8337 | 28,1% | 1000 | | 34,8% | 5216 | | 37,4% | 479 | | 42,1% | | 3121 | 19,9% | 521 | 30,0% |
|  | *Obese* | 2957 | 10,0% | 491 | | 17,1% | 1521 | | 10,9% | 181 | | 15,9% | | 1436 | 9,2% | 310 | 17,8% |
| Smoking Habit | *Non-smoker* | 22787 | 76,9% | 2346 | | 81,5% | 10006 | | 71,7% | 875 | | 76,9% | | 12781 | 81,5% | 1471 | 84,6% |
|  | *Current-smoker* | 6857 | 23,1% | 531 | | 18,5% | 3948 | | 28,3% | 263 | | 23,1% | | 2909 | 18,5% | 268 | 15,4% |
| Alcohol intake | *None* | 7720 | 26,0% | 1511 | | 52,5% | 1855 | | 13,3% | 500 | | 43,9% | | 5865 | 37,4% | 1011 | 58,1% |
|  | *1-2* | 10703 | 36,1% | 737 | | 25,6% | 4395 | | 31,5% | 247 | | 21,7% | | 6308 | 40,2% | 490 | 28,2% |
|  | *3+* | 11221 | 37,9% | 629 | | 21,9% | 7704 | | 55,2% | 391 | | 34,4% | | 3517 | 22,4% | 238 | 13,7% |
| Education | *Elementary/Middle* | 8200 | 27,7% | 1402 | | 48,7% | 3952 | | 28,3% | 633 | | 55,6% | | 4248 | 27,1% | 769 | 44,2% |
|  | *Highschool* | 15518 | 52,3% | 1200 | | 41,7% | 7704 | | 55,2% | 436 | | 38,3% | | 7814 | 49,8% | 764 | 43,9% |
|  | *University* | 5926 | 20,0% | 275 | | 9,6% | 2298 | | 16,5% | 69 | | 6,1% | | 3628 | 23,1% | 206 | 11,8% |
| Salt | *Low* | 13378 | 45,1% | 853 | | 29,6% | 5725 | | 41,0% | 319 | | 28,0% | | 7653 | 48,8% | 534 | 30,7% |
|  | *Medium* | 14664 | 49,5% | 1759 | | 61,1% | 7323 | | 52,5% | 729 | | 64,1% | | 7341 | 46,8% | 1030 | 59,2% |
|  | *High* | 1602 | 5,4% | 265 | | 9,2% | 906 | | 6,5% | 90 | | 7,9% | | 696 | 4,4% | 175 | 10,1% |
| Sweets | *1* | 10358 | 34,9% | 1074 | | 37,3% | 5124 | | 36,7% | 428 | | 37,6% | | 5234 | 33,4% | 646 | 37,1% |
|  | *2* | 8486 | 28,6% | 762 | | 26,5% | 3757 | | 26,9% | 260 | | 22,8% | | 4729 | 30,1% | 502 | 28,9% |
|  | *3* | 10800 | 36,4% | 1041 | | 36,2% | 5073 | | 36,4% | 450 | | 39,5% | | 5727 | 36,5% | 591 | 34,0% |
| meat | *1* | 13483 | 45,5% | 1368 | | 47,5% | 5143 | | 36,9% | 468 | | 41,1% | | 8340 | 53,2% | 900 | 51,8% |
|  | *2* | 5301 | 17,9% | 469 | | 16,3% | 2508 | | 18,0% | 179 | | 15,7% | | 2793 | 17,8% | 290 | 16,7% |
|  | *3* | 10860 | 36,6% | 1040 | | 36,1% | 6303 | | 45,2% | 491 | | 43,1% | | 4557 | 29,0% | 549 | 31,6% |
| **Outcomes** | |  | | | | | | | | | | | | | | | |
|  | | *Mean (SD)* | *Min-Max* | *Mean (SD)* | | *Min-Max* | *Mean (SD)* | | *Min-Max* | *Mean (SD)* | | *Min-Max* | | *Mean (SD)* | *Min-Max* | *Mean (SD)* | *Min-Max* |
| systolic blood pressure | | 121.8 (14.7) | 70; 243 | 120.5 (14.6) | | 70; 220 | 126.4 (13.7) | | 70; 243 | 125.2 (14.1) | | 75; 220 | | 117.7 (14.3) | 80; 220 | 117.5 (14.2) | 75; 205.5 |
| diastolic blood pressure | | 76.8 (10.0) | 25; 148 | 76.14 (10.2) | | 25; 130 | 79.2 (9.7) | | 40; 148 | 79.2 (9.7) | | 45; 115 | | 74.8 (9.8) | 45; 115 | 74.5 (10.0) | 45; 110 |
|  |  | **ITALY (n=34,131)** | | **HMPC (n=3,249)** | | | **ITALY (n=16,493)** | | | **HMPC (n=1,317)** | | | | **ITALY (n=17,638)** | | **HMPC (n=1,932)** | |
|  |  | *N* | *%* | *N* | | *%* | *N* | | *%* | *N* | | *%* | | *N* | *%* | *N* | *%* |
| hypertension prevalence (n= 37,380) | | 8643 | 25.3% | 710 | | 21.9% | 5380 | | 32.6% | 374 | | 28.4% | | 3263 | 18.5% | 336 | 17.4% |

Table S2. Characteristics of the subgroups by macro-area of origin: Asia, North Africa and Central-Eastern Europe

| **Characteristics** | | **Asia (n=508)** | | **North Africa (n=608)** | | **CE Europe (n=1,426)** | |
| --- | --- | --- | --- | --- | --- | --- | --- |
|  |  |  |  |  |  |  |  |
| **Covariates** | |  |  |  |  |  |  |
| **continuous variables** | | *Mean (SD)* | *Min-Max* | *Mean (SD)* | *Min-Max* | *Mean (SD)* | *Min-Max* |
| Age (years) | | 38.3 (9.4) | 20; 65 | 40.0 (9.2) | 20; 64 | 39.3 (9.6) | 20; 66 |
| BMI (kg/m2) | | 25.5 (4.3) | 16.0; 45.7 | 26.9 (4.6) | 15.6; 52.6 | 25.5 (4.6) | 15.6; 51.6 |
| **categorical variables** | | *N* | *%* | *N* | *%* | *N* | *%* |
| Sex | *Females* | 271 | 53.3% | 323 | 53.1% | 951 | 66.7% |
|  | *Males* | 237 | 46.7% | 285 | 46.9% | 475 | 33.3% |
| BMI | *Normal weight* | 243 | 47.8% | 239 | 39.3% | 727 | 51.0% |
|  | *Overweight* | 186 | 36.6% | 224 | 36.8% | 485 | 34.0% |
|  | *Obese* | 79 | 15.6% | 145 | 23.8% | 214 | 15.0% |
| Smoking Habit | *Non-smoker/Previous* | 458 | 90.2% | 544 | 89.5% | 1058 | 74.2% |
|  | *Current-smoker* | 50 | 9.8% | 64 | 10.5% | 368 | 25.8% |
| Alcohol intake | *None* | 364 | 71.7% | 573 | 94.2% | 426 | 29.9% |
|  | *1-2* | 79 | 15.6% | 19 | 3.1% | 541 | 37.9% |
|  | *3+* | 65 | 12.8% | 16 | 2.6% | 459 | 32.2% |
| Education | *Elementary/Middle* | 305 | 60.0% | 449 | 73.8% | 497 | 34.9% |
|  | *Highschool* | 155 | 30.5% | 121 | 19.9% | 786 | 55.1% |
|  | *University* | 48 | 9.4% | 38 | 6.3% | 143 | 10.0% |
| salt | *Low* | 116 | 22.8% | 187 | 30.8% | 423 | 29.7% |
|  | *Medium* | 357 | 70.3% | 369 | 60.7% | 856 | 60.0% |
|  | *High* | 35 | 6.9% | 52 | 8.6% | 147 | 10.3% |
| sweets | *1* | 211 | 41.5% | 213 | 35.0% | 507 | 35.6% |
|  | *2* | 124 | 24.4% | 171 | 28.1% | 388 | 27.2% |
|  | *3* | 173 | 34.1% | 224 | 36.8% | 531 | 37.2% |
| meat | *1* | 389 | 76.6% | 263 | 43.3% | 570 | 40.0% |
|  | *2* | 57 | 11.2% | 94 | 15.5% | 263 | 18.4% |
|  | *3* | 62 | 12.2% | 251 | 41.3% | 593 | 41.6% |
| **Outcomes** | | | | | | | |
|  | | *Mean (SD)* | *Min-Max* | *Mean (SD)* | *Min-Max* | *Mean (SD)* | *Min-Max* |
| systolic blood pressure | | 120.4 (15.2) | 80; 220 | 121.7 (14.1) | 80; 196 | 120 (14.5) | 75; 185 |
| diastolic blood pressure | | 76.2 (10.3) | 50; 110 | 75.8 (9.6) | 45; 104 | 76.3 (10.2) | 45; 111 |
|  |  | **Asia (n=576)** | | **North Africa (n=652)** | | **CE Europe (n=1,628)** | |
|  |  | *N* | *%* | *N* | *%* | *N* | *%* |
| hypertension prevalence (n=37,380) | | 127 | 22,0% | 108 | 16,6% | 380 | 23,3% |

Table S3. Multiple mediation analysis for immigrants from the 3 macroareas (Asia, North Africa, CE Europe) vs Italy

|  | **SYSTOLIC BLOOD PRESSURE (β 95% CI)** | | | **HYPERTENSION (OR 95% CI)** | | |
| --- | --- | --- | --- | --- | --- | --- |
|  | **Asia vs Italy** | **North Africa vs Italy** | **CE Europe vs Italy** | **Asia vs Italy** | **North Africa vs Italy** | **CE Europe vs Italy** |
| *Total Effect* | -1.08  (-2.43; 0.06) | 0.00  (-1.29; 1.14) | -0.90  (-1.38; 0.24) | 0.95  (0.80; 1.24) | 0.61  (0.48; 0.79) | 1.00  (0.89; 1.14) |
| *Total Direct Effect* | -1.40  (-2.48; -0.28) | -1.65  (-3.07; -0.36) | -1.81  (-2.32; -0.83) | 0.87  (0.73; 1.14) | 0.47  (0.36; 0.59) | 0.84  (0.75; 0.96) |
| *Total Indirect Effect* | 0.31  (-0.31; 0.69) | 1.65  (1.09; 2.19) | 0.91  (0.83; 1.17) | 1.09  (1.03; 1.18) | 1.31  (1.24; 1.44) | 1.19  (1.15; 1.24) |
| *BMI* | 1.00  (0.55; 1.38) | 2.11  (1.8; 2.59) | 0.90  (0.79; 1.09) | 1.13  (1.09; 1.20) | 1.37  (1.30; 1.44) | 1.18  (1.14; 1.22) |
| *Alcohol* | -0.48  (-0.63; -0.35) | -0.71  (-0.99; -0.45) | -0.05  (-0.09; 0.00) | 0.96  (0.94; 0.99) | 0.94  (0.92; 0.99) | 0.99  (0.99; 1.00) |
| *Education* | 0.13  (0.00; 0.25) | 0.16  (-0.02; 0.33) | 0.04  (0.00; 0.09) | 1.02  (1.00; 1.03) | 1.02  (0.99; 1.04) | 1.02  (1.01; 1.03) |
| *Sweets* | -0.05  (-0.10; -0.02) | 0.00  (-0.04; 0.04) | 0.00  (-0.03; 0.03) | 1.01  (1.00; 1.01) | 1.00  (1.00; 1.01) | 1.00  (1.00; 1.00) |
| *Meat* | -0.34  (-0.47; -0.24) | 0.03  (-0.03; 0.09) | 0.06  (0.02; 0.09) | 0.98  (0.96; 0.99) | 1.00  (1.00; 1.01) | 1.00  (1.00; 1.01) |

(The sum of the percent explained effects of the individual mediators may not equal the total indirect effect because of correlation and overlapping mediation effects among mediators that is reflected in the total indirect effect but not the individual mediators)

*Table S4. Sensitivity analysis: Multiple Mediation Analysis for HMPC vs Italy, all covariates included, stratified by gender*

|  | **Systolic Blood Pressure** | | | **Hypertension** | | |
| --- | --- | --- | --- | --- | --- | --- |
|  | **Total β (95% CI)** | **Males β (95% CI)** | **Females β (95% CI)** | **Total OR (95% CI)** | **Males OR (95% CI)** | **Females OR (95% CI)** |
| *Total Effect* | -0.6  (-1.24; 0.1) | -1.23  (-2.15; -0.57) | 0.12  (-0.62; 0.85) | 0.91  (0.80; 1.01) | 0.79  (0.68; 0.90) | 1.14  (1.00; 1.31) |
| *Total Direct Effect* | -1.53  (-2.1; -0.91) | -1.52  (-2.16; -0.95) | -1.25  (-2.00; -0.55) | 0.78  (0.69; 0.85) | 0.76  (0.64; 0.86) | 0.88  (0.76; 1.01) |
| *Total Indirect Effect* | 0.93  (0.69; 1.18) | 0.29  (-0.19; 0.57) | 1.37  (1.20; 1.57) | 1.17  (1.12; 1.23) | 1.05  (1.00; 1.10) | 1.30  (1.22; 1.39) |
| *BMI* | 1.22  (1.06; 1.32) | 0.88  (0.50; 1.11) | 1.61  (1.47; 1.78) | 1.20  (1.16; 1.24) | 1.12  (1.07; 1.16) | 1.30  (1.24; 1.37) |
| *Smoke* | 0.01  (-0.01; 0.03) | 0.01  (-0.02; 0,04) | 0.02  (0.00; 0.03) | 1.00  (1.00; 1.00) | 1.00  (1.00; 1.00) | 1.00  (1.00; 1.00) |
| *Alcohol* | -0.30  (-0.41; -0.18) | -0.51  (-0.65; -0.35) | -0.21  (-0.29; -0.18) | 0.97  (0.96; 0.99) | 0.94  (0.92; 0.97) | 0.99  (0.97; 1.00) |
| *Education* | 0.10  (0.03; 0.17) | 0.05  (-0.05; 0.07) | 0.10  (0.00; 0.15) | 1.01  (1.00; 1.03) | 1.00  (0.98; 1.02) | 1.02  (1.00; 1.04) |
| *Salt* | -0.05  (-0.09; 0.00) | -0.05  (-0.10; -0.03) | -0.04  (-0.17; 0.06) | 0.99  (0.99; 1.00) | 0.99  (0.98; 1.00) | 0.99  (0.98; 1.01) |
| *Sweets* | -0.02  (-0.03; 0.01) | 0.01  (-0.01; 0.03) | -0.06  (-0.10; 0.00) | 1.00  (1.00; 1.00) | 1.00  (1.00; 1.00) | 1.00  (1.00; 1.00) |
| *Meat* | -0.02  (-0.03; 0.00) | -0.05  (-0.11; -0.01) | 0.01  (-0.01; 0.04) | 1.00  (1.00; 1.00) | 1.00  (1.00; 1.00) | 1.00  (1.00; 1.00) |

(The sum of the percent explained effects of the individual mediators may not equal the total indirect effect because of correlation and overlapping mediation effects among mediators that is reflected in the total indirect effect but not the individual mediators)

Table S5. Sensitivity analysis: Multiple Mediation Analysis for HMPC vs Italy, all covariates included, stratified by area of origin

|  | **SYSTOLIC BLOOD PRESSURE (β 95% CI)** | | | **HYPERTENSION (OR 95% CI)** | | |
| --- | --- | --- | --- | --- | --- | --- |
|  | **Asia vs Italy** | **North Africa vs Italy** | **CE Europe vs Italy** | **Asia vs Italy** | **North Africa vs Italy** | **CE Europe vs Italy** |
| *Total Effect* | -1.01  (-2.52; 0.16) | -0.11  (-1.16; 0.91) | -0.82  (-1.47; -0.16) | 0.97  (0.74; 1.18) | 0.62  (0.53; 0.76) | 1.00  (0.90; 1.11) |
| *Total Direct Effect* | -1.32  (-2.68; 0.07) | -1.62  (-2.71; -0.75) | -1.73  (-2.38; -1.09) | 0.90  (0.68; 1.09) | 0.47  (0.40; 0.59) | 0.84  (0.76; 0.94) |
| *Total Indirect Effect* | 0.31  (-0.34; 0.58) | 1.51  (1.15; 2.06) | 0.91  (0.66; 1.18) | 1.08  (1.02; 1.15) | 1.31  (1.21; 1.40) | 1.19  (1.14; 1.22) |
| *BMI* | 1.03  (0.66; 1.19) | 2.12  (1.74; 2.53) | 0.92  (0.69; 1.14) | 1.15  (1.08; 1.21) | 1.38  (1.28; 1.43) | 1.18  (1.13; 1.22) |
| *Smoke* | 0.04  (-0.01; 0.10) | 0.03  (0.01; 0.08) | -0.01  (-0.02; 0.00) | 1.00  (0.99; 1.01) | 1.00  (0.99; 1.01) | 1.00  (1.00; 1.00) |
| *Alcohol* | -0.51  (-0.67; -0.36) | -0.75  (-1.02; -0.48) | -0.05  (-0.08; -0.01) | 0.96  (0.93; 1.00) | 0.94  (0.91; 0.99) | 0.99  (0.99; 1.00) |
| *Education* | 0.15  (0.01; 0.24) | 0.18  (0.02; 0.36) | 0.04  (0.01; 0.08) | 1.02  (1.00; 1.04) | 1.02  (0.99; 1.05) | 1.02  (1.01; 1.02) |
| *Salt* | -0.04  (-0.12; 0.03) | -0.04  (-0.11; 0.02) | -0.06  (-0.11; -0.01) | 0.99  (0.98; 1.00) | 0.99  (0.98; 1.00) | 0.99  (0.98; 1.00) |
| *Sweets* | -0.05  (-0.1; -0.01) | 0.00  (-0.04; 0.05) | -0.01  (-0.02; 0.02) | 1.00  (1.00; 1.01) | 1.00  (1.00; 1.01) | 1.00  (1.00; 1.00) |
| *Meat* | -0.36  (-0.48; -0.23) | 0.04  (0.00; 0.08) | 0.06  (0.04; 0.1) | 0.98  (0.96; 0.99) | 1.00  (1.00; 1.01) | 1.00  (1.00; 1.01) |

(The sum of the percent explained effects of the individual mediators may not equal the total indirect effect because of correlation and overlapping mediation effects among mediators that is reflected in the total indirect effect but not the individual mediators)

Table S6. Number and percentage of subjects from each country of birth in each macro-area of origin

| **CENTRAL-EASTERN EUROPE** | **N** | **%** |
| --- | --- | --- |
| ROMANIA | 568 | 34,9% |
| ALBANIA | 398 | 24,4% |
| SERBIA | 277 | 17,0% |
| MOLDAVIA | 125 | 7,7% |
| POLAND | 64 | 3,9% |
| UKRAINE | 43 | 2,6% |
| BULGARIA | 37 | 2,3% |
| BOSNIA | 36 | 2,2% |
| RUSSIA | 14 | 0,9% |
| BELARUS | 10 | 0,6% |
| SLOVAK REPUBLIC | 10 | 0,6% |
| CROATIA | 9 | 0,6% |
| HUNGARY | 7 | 0,4% |
| CZECH REPUBLIC | 6 | 0,4% |
| MACEDONIA | 6 | 0,4% |
| YUGOSLAVIA | 5 | 0,3% |
| LATVIA | 3 | 0,2% |
| LITHUANIA | 3 | 0,2% |
| KOSOVO | 2 | 0,1% |
| SLOVENIA | 2 | 0,1% |
| ESTONIA | 1 | 0,1% |
| MONTENEGRO | 1 | 0,1% |
| OTHER | 1 | 0,1% |
| **SUB-SAHARAN AFRICA** | **N** | **%** |
| IVORY COAST | 50 | 25,5% |
| SENEGAL | 44 | 22,4% |
| NIGERIA | 32 | 16,3% |
| GHANA | 28 | 14,3% |
| ETHIOPIA | 9 | 4,6% |
| BURKINA FASO | 7 | 3,6% |
| GUINEA | 4 | 2,0% |
| KENYA | 3 | 1,5% |
| TOGO | 3 | 1,5% |
| BURUNDI | 2 | 1,0% |
| CHAD | 2 | 1,0% |
| GUINEA BISSAU | 2 | 1,0% |
| SOUTH AFRICA | 2 | 1,0% |
| RWANDA | 2 | 1,0% |
| CAMEROON | 1 | 0,5% |
| CONGO | 1 | 0,5% |
| GABON | 1 | 0,5% |
| MADAGASCAR | 1 | 0,5% |
| MALI | 1 | 0,5% |
| ZAMBIA | 1 | 0,5% |
| **NORTHERN AFRICA** | **N** | **%** |
| MOROCCO | 630 | 96,6% |
| TUNISIA | 10 | 1,5% |
| EGYPT | 5 | 0,8% |
| ALGERIA | 4 | 0,6% |
| LIBYA | 3 | 0,5% |
| **ASIA** | **N** | **%** |
| INDIA | 393 | 68,2% |
| BANGLADESH | 100 | 17,4% |
| CHINA | 43 | 7,5% |
| GEORGIA | 14 | 2,4% |
| SRI LANKA | 7 | 1,2% |
| LEBANON | 3 | 0,5% |
| PHILIPPINES | 2 | 0,3% |
| IRAN | 2 | 0,3% |
| KAZAKHSTAN | 2 | 0,3% |
| NEPAL | 2 | 0,3% |
| SYRIA | 2 | 0,3% |
| THAILAND | 2 | 0,3% |
| ARMENIA | 1 | 0,2% |
| KYRGYZSTAN | 1 | 0,2% |
| PAKISTAN | 1 | 0,2% |
| UZBEKISTAN | 1 | 0,2% |
| **CENTRAL-SOUTHERN AMERICA** | **N** | **%** |
| BRAZIL | 65 | 33,0% |
| ARGENTINA | 40 | 20,3% |
| PERU | 20 | 10,2% |
| COLOMBIA | 12 | 6,1% |
| CUBA | 12 | 6,1% |
| DOMINICAN REPUBLIC | 12 | 6,1% |
| VENEZUELA | 11 | 5,6% |
| GUATEMALA | 6 | 3,0% |
| CHILE | 5 | 2,5% |
| PARAGUAY | 5 | 2,5% |
| MEXICO | 3 | 1,5% |
| ECUADOR | 2 | 1,0% |
| BOLIVIA | 1 | 0,5% |
| COSTA RICA | 1 | 0,5% |
| HONDURAS | 1 | 0,5% |
| NICARAGUA | 1 | 0,5% |
